# Successful endoscopic closure of delayed perforation using hemoclips after colorectal endoscopic submucosal dissection

**DOI:** 10.1055/a-2494-9982

**Published:** 2025-01-14

**Authors:** Nao Takeuchi, Ken Ohata, Yuki Kano, Kohei Ono, Ryoju Negishi, Yohei Minato, Hideyuki Chiba

**Affiliations:** 1 13635 Gastrointestinal Endoscopy, NTT Medical Center Tokyo, Shinagawa-ku, Japan; 2 215674 Digestive Disease Center, Itabashi Chuo Medical Center, Itabashi-ku, Japan; 3 74155 Gastroenterology, Omori Red Cross Hospital, Ota-ku, Japan

Delayed perforation following colorectal endoscopic submucosal dissection (ESD) occurs in approximately 0.4% of cases 1 2 . Although it is rare, emergency surgery is required in nearly half of these cases 3 . Endoscopic closure for such perforations is rarely performed. No cases of successful closure of delayed perforation using hemoclips after colorectal ESD have yet been reported. Here, we present a case of delayed perforation following colon ESD that was successfully closed with hemoclips and managed conservatively.

A 44-year-old woman underwent ESD for a 45-mm tumor located in the ascending colon ( Fig. 1 ). The tumor was resected en bloc without complications. However, 18 h post-procedure, she developed a fever of 38°C and experienced localized right lower abdominal pain without rebound tenderness. She was initially managed with fasting, but her fever and abdominal pain persisted until day 3. Computed tomography (CT) revealed minimal extraluminal air and fluid near the ESD site, suggestive of a micro-perforation ( Fig. 2 ). Considering the mild symptoms and CT findings, emergency endoscopy was planned after consultation with surgeons.

**Fig. 1:**
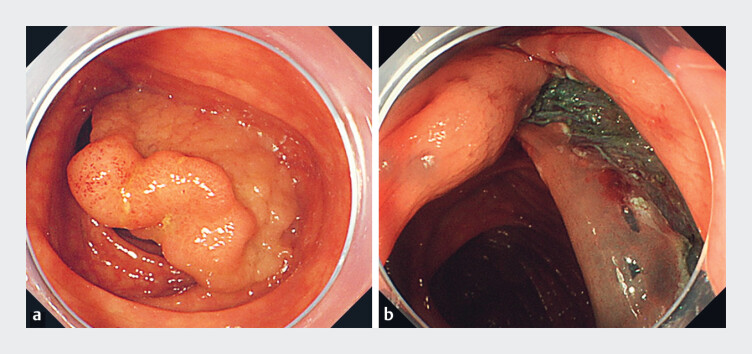
Endoscopic view. **a** Endoscopic view of tumor before endoscopic submucosal dissection (ESD). **b** No perforation or thermal damage was noted after ESD.

**Fig. 2:**
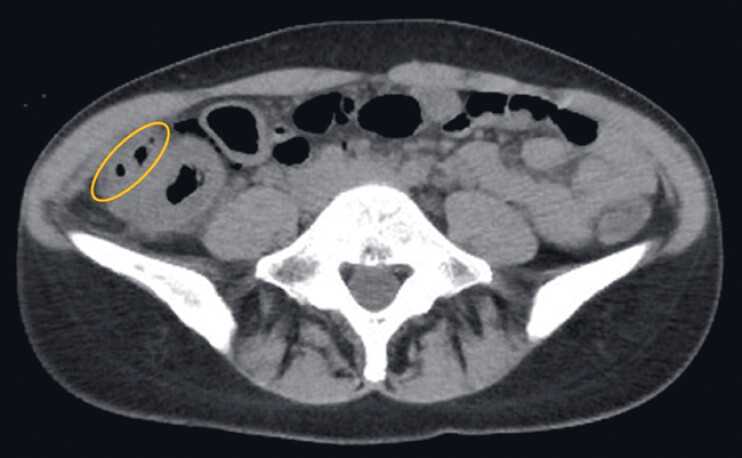
Computed tomography showing a small amount of extraintestinal air (yellow oval) around the ulcer site after endoscopic submucosal dissection.

Colonoscopy, performed without prior bowel preparation, revealed two 5-mm perforation sites. These were closed using reopenable clips, reinforced with the less expensive hemoclips ( Fig. 3 , Video 1 ). Following closure, the patient’s abdominal pain resolved promptly. Oral intake was resumed on day 6, and she was discharged on day 8. No recurrence of pain or abscess formation was noted after closure of the perforation sites.

**Fig. 3:**
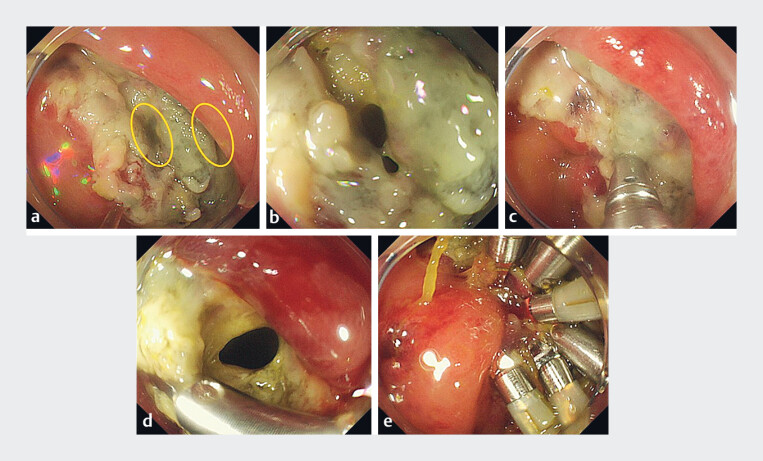
Closure of the perforation sites with hemoclips. **a** Two perforation sites (yellow ovals) were found in the defect created after endoscopic submucosal dissection. **b, c** The first perforation site was closed with a re-openable clip. **d** The second perforation site is seen. **e** Both perforation sites were completely closed using hemoclips.

Successful endoscopic closure of delayed perforation using hemoclips after colorectal endoscopic submucosal dissection.Video 1

Kuwabara et al. previously reported a case of delayed perforation after hybrid ESD that was successfully closed using an over-the-scope (OTS) clip system 4 . While the OTS clip offers robust closure, its application can be challenging in cases involving multiple perforations, as it may obstruct additional clip placements. Furthermore, large ulcers pose a risk of tearing the ulcer bed during OTS clip deployment. High costs and the inability to reposition the clip further limit its utility.

In the present case, the large ulcer size and the presence of two perforation sites made hemoclips a more suitable choice. Hemoclips provided effective closure while maintaining cost efficiency and avoiding potential complications associated with the OTS clip system.

Although the use of hemoclips is context-dependent, when the perforation site is identifiable and accessible, closure with hemoclips may avoid the need for emergency surgery at a lower cost compared to OTS clips.

Endoscopy_UCTN_Code_CPL_1AJ_2AD_3AD

## Notes

Endoscopy E-Videos https://eref.thieme.de/e-videos
*E-Videos* is an open access online section of the journal *Endoscopy* , reporting on interesting cases and new techniques in gastroenterological endoscopy. All papers include a high-quality video and are published with a Creative Commons CC-BY license. Endoscopy E-Videos qualify for HINARI discounts and waivers and eligibility is automatically checked during the submission process. We grant 100% waivers to articles whose corresponding authors are based in Group A countries and 50% waivers to those who are based in Group B countries as classified by Research4Life (see: https://www.research4life.org/access/eligibility/ ). This section has its own submission website at https://mc.manuscriptcentral.com/e-videos .
